# Ontogenetic development of the auditory sensory organ in zebrafish (*Danio rerio*): changes in hearing sensitivity and related morphology

**DOI:** 10.1038/srep15943

**Published:** 2015-11-03

**Authors:** Jiping Wang, Qiang Song, Dongzhen Yu, Guang Yang, Li Xia, Kaiming Su, Haibo Shi, Jian Wang, Shankai Yin

**Affiliations:** 1 Department of Otolaryngology, Affiliated Sixth People’s Hospital of Shanghai Jiao Tong University, Otolaryngology Institute of Shanghai Jiao Tong University, Shanghai 200233, China; 2 School of Human Communication Disorder, Dalhousie University, Halifax, Nova Scotia, Canada

## Abstract

Zebrafish (*Danio rerio*) is an important model organism in hearing research. However, data on the hearing sensitivity of zebrafish vary across different reports. In the present study, the hearing sensitivity of zebrafish was examined by analysing the auditory evoked potentials (AEPs) over a range of total lengths (TLs) from 12 to 46 mm. Morphological changes in the hair cells (HCs) of the saccule (the main auditory end organ) and their synapses with primary auditory neurons were investigated. The AEPs were detected up to a much higher frequency limit (12 kHz) than previously reported. No significant difference in the frequency response range was observed across the TL range examined. However, the AEP thresholds demonstrated both developmental improvement and age-related loss of hearing sensitivity. The changes in hearing sensitivity were roughly consistent with the morphological changes in the saccule including (1) the number and density of HCs, (2) the organization of stereocilia, and (3) the quantity of a main ribbon protein, Ribeye b. The results of this study established a clear baseline for the hearing ability of zebrafish and revealed that the changes in the saccule contribute to the observed changes in TL (age)-related hearing sensitivity.

The zebrafish (*Danio rerio*), which has become an important vertebrate model organism, is a potentially powerful tool for evaluating hearing genetics, the mechanisms involved in hair cell (HC) regeneration and medical screening for the treatment of auditory dysfunction^1^,^2^,^3^. Despite its increasing importance as an auditory model, studies on the hearing ability and hearing development in zebrafish have lagged behind those in other model systems.

In 1993, Platt^4^ described the structure of the adult zebrafish inner ear and found that it was similar to that of goldfish in terms of gross structure, the otolith shape, macular shape, HC orientation pattern and so on. A few years later (1996), a detailed description of the inner ear of zebrafish embryos aged 0–7 days post-fertilization (dpf) was reported by Haddon and Lewis^5^. That report provided a foundation in the understanding of the development of the otic vesicle, otoliths and HCs in this species. In 2001, another study^6^ of juvenile and adult zebrafish added new details regarding inner ear development. Zebrafish, similar to goldfish, belong to a category termed “hearing specialists”. Fishes in this group have an accessory auditory structure, the Weberian apparatus (or ossicles), which communicates acoustic signals from the swim bladders to the auditory sensory organ (the inner ear), primarily to the saccule^7^. In 2004, the ontogeny of the Weberian apparatus was investigated by Grande and Young^8^ in zebrafish ranging from 3 to 28 mm in total length (TL). This study indicated that Weberian ossicles and swim bladders become positioned to transmit sound in zebrafish at a TL between 7.5 and 18.0 mm^7^,^9^. In addition to these important morphological studies, several other studies on the hearing ability of zebrafish have been reported but have varied in terms of their methodologies and findings^10^,^11^,^12^,^13^,^14^,^15^. In the early 2000s, Higgs *et al.* analysed hearing in zebrafish by recording auditory evoked potentials (AEPs) in two studies^10^,^11^. Their first study suffered from a sound calibration errors, and the responses reported in these two papers generally were noisy (AEP waveforms with a higher noise floor were presented in the articles), this noise was likely due to magnetic contaminations of the testing field. The second study reported an age-related expansion of the maximal detectable response frequency from 200 Hz in the youngest fish, with a TL of 10 mm to 4 kHz in fish with a TL of 25–35 mm; no further expansion was observed in larger fish. The authors attributed this frequency expansion to the development of the Weberian apparatus but claimed that there were no developmental changes in the AEP threshold, amplitude or latency^11^. In contrast to these electrophysiological observations, Zeddies and Fay^12^ found no change in the hearing frequency range of zebrafish from 5 dpf to adulthood based on a startle response experiment. Notably, the hearing frequency thresholds in this behavioural study were much higher than those obtained by measuring AEPs. In another behavioural assay using a positive reinforcement paradigm^13^, Higgs *et al.* reported higher thresholds than those obtained from their AEP data (for example at 600 Hz, they observed 115 dBW for the behavioural test and 100 dBw for the AEP assessment)^10^,^11^. Unfortunately, in those two behavioural reports^12^,^13^, the tests did not measure beyond 2 kHz and the latter report by Higgs *et al.* focused on zebrafish from only 28 to 36 mm TL. More recently, two studies investigated hearing ability in larval zebrafish (before 7 dpf) by observing microphonic potential^14^ and behavioural prepulse inhibition^15^. These studies revealed decreases in the hearing threshold during the first week of life in zebrafish. However, it is impossible to compare these two most recent studies with previous reports because they used very different methods.

In summary, to date, there is no clear, accepted baseline AEP profile for zebrafish hearing according to their ontogenetic development, although such data are available for many other species^16^,^17^,^18^,^19^,^20^,^21^,^22^,^23^,^24^. Additionally, no data are available regarding the hearing change in this species with old age for age-related hearing loss, Furthermore, except for the developmental changes in the Weberian apparatus, it is unclear whether any other structural changes may be responsible for the hearing changes during either ontogenetic development or ageing.

Because AEPs are a sensitive and widely used measure of fish hearing^25^,^26^,^27^,^28^, we attempted to establish a baseline hearing profile in zebrafish over a large age range, from 40 dpf (TL  = 12 mm) to 20 months post-fertilization (TL = 46 mm). In this study, zebrafish greater than 16 months old (TL > 42 mm) were assigned to an “old age” group considering the 2-year lifespan of this species, as observed in our lab. Surprisingly, we found a much broader hearing frequency range (up to 12 kHz) in our sample than that which has been reported by others, and the response frequencies did not significantly change within the age span observed. However, a clear TL (age)-related change in hearing sensitivity was detected.

Due to the role of the Weberian apparatus in the hearing of “hearing specialists”, most researchers consider the saccule as the major auditory organ, although there are data indicating a role of the lagena in hearing^29^,^30^. Therefore, we focused on changes in the saccule in the present study. Morphological changes surrounding saccular HCs were examined to verify the contributors to age-related hearing sensitivity changes.

## Results

### Auditory evoked potentials and hearing sensitivity

**Figure 1:**
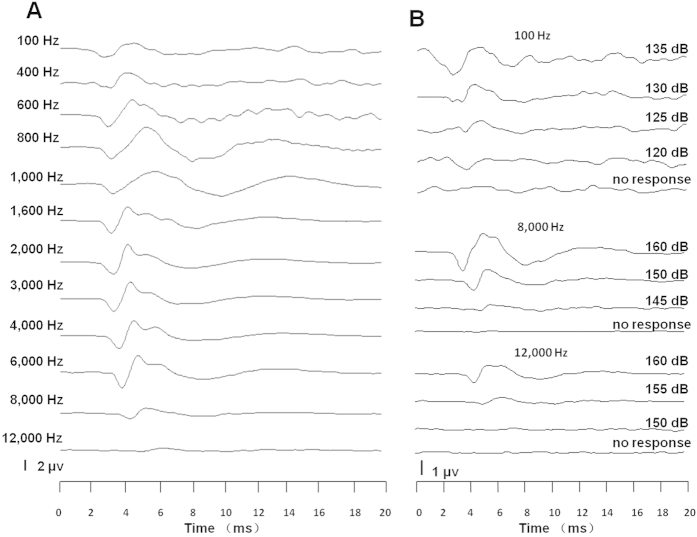
Typical AEP waveforms from a 35 mm zebrafish. (**A**) AEPs evoked by tone bursts of different frequencies. The sound intensities were 140 dBw for tone bursts from 100 Hz to 6 kHz, 150 dBw for 8 kHz and 155 dBw for 12 kHz. (**B**) AEP threshold tracking at 100 Hz (upper panel), 8 kHz (middle panel) and 12 kHz (lower panel).

We examined the hearing ability of zebrafish using frequency-specific AEPs. To establish the reliable recording of auditory responses, we used various tests to confirm that the waveforms were auditory responses, not artefacts (see Supplementary Figs S1–4). The first significant finding of the present study was that the hearing frequency response range of the zebrafish that we recorded were much broader than that which has previously been reported, especially at the high frequency end^10^. Figure 1A shows the AEP waveforms recorded from an adult 35 mm zebrafish evoked by tone bursts of distinct frequencies. This fish clearly responded to sound from 100 Hz to 12 kHz, although a higher sound level was required towards the high frequency end (150 or 155 dB per μPa, abbreviated as dBw in the remainder of the text). Figure 1B shows the tracking of response thresholds from this subject at 3 different frequencies.

**Figure 2:**
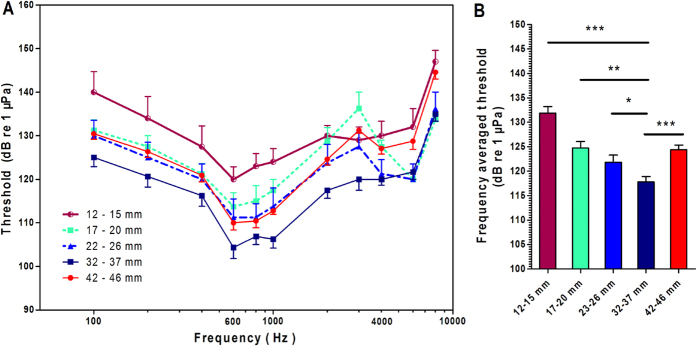
AEP threshold comparison across five TL groups. (**A**) AEP threshold audiogram (between 100 Hz and 8 kHz). (**B**) Frequency-averaged thresholds across groups. *P <  0.05, **P < 0.01, ***P < 0.001, Tukey’s *post hoc* tests between individual groups after a one-way ANOVA for the effect of TL. All data in this report are presented as the means ± SEM.

We measured AEP thresholds across 11 frequencies between 100 Hz and 8 kHz in five TL groups (TL = 12–15 mm, n = 6; TL = 17–20 mm, n = 4; TL = 22–26 mm, n = 4; TL = 32–37 mm, n = 9; and TL = 42–46 mm, n = 12; see Table 1 for corresponding age information). The responses at 12 kHz were omitted because they were evoked only at sound levels greater than 150 dBw. Figure 2A shows the AEP audiogram of the different TL groups. Overall, there was a trend towards a decrease in the hearing frequency threshold from the smallest TL group to the group with TL = 32–37 mm, followed by an increase in the hearing frequency threshold in the largest TL group. These results demonstrated both developmental improvement and age-related loss of hearing sensitivity. All five groups showed best hearing abilities at frequencies between 600 and 1,000 Hz. The lowest AEP threshold was at 600 Hz in the group with a TL of 32–37 mm (104.38 ± 2.58 dBw, Supplementary Table S1). To simplify the age-related changes in hearing sensitivity, the frequency threshold data presented in Fig. 2A were converted to frequency-averaged thresholds, which are shown in Fig. 2B. The mean values of the frequency-averaged thresholds were (mean ± SEM) 141.7 ± 1.32, 124.8 ± 1.31, 121.8 ± 1.49, 117.8 ± 1.09 and 124.4 ± 1.87 dBw, respectively, for the 5 TL groups identified above. One-way ANOVA showed a significant effect of TL on hearing sensitivity (*F*_4_,_368_ = 54.64, *P* < 0.001). *Post hoc* pairwise tests showed significant differences between these groups, as summarized in Fig. 2B.

### Developmental changes in saccular HCs

**Figure 3:**
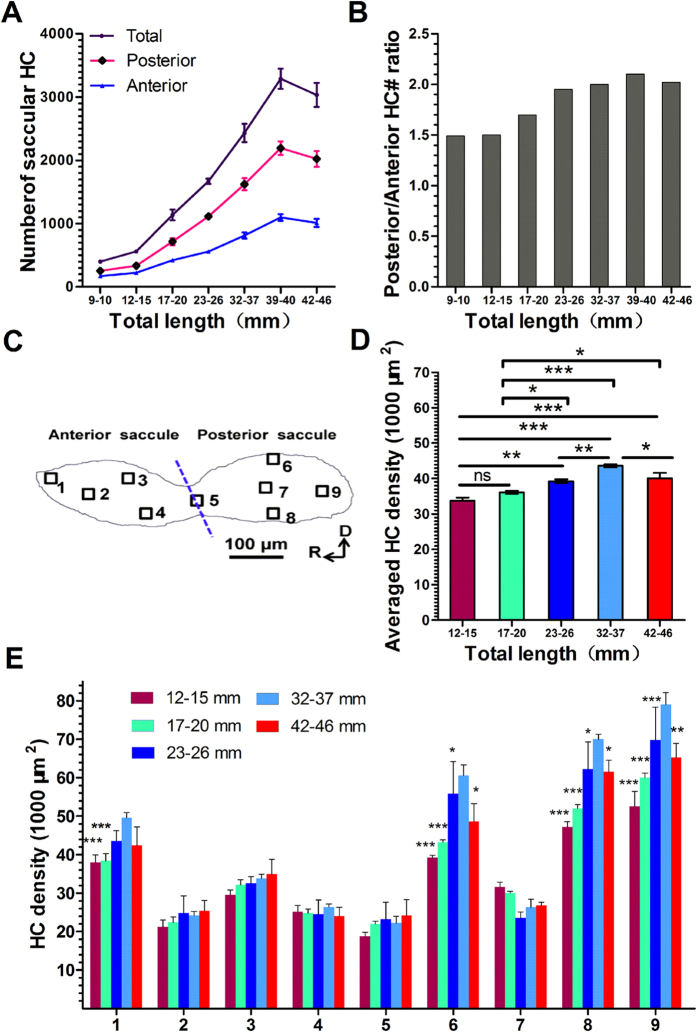
Developmental changes in HC number and density. (**A**) Differences in HC number according to TL for the total (including posterior and anterior) and separated posterior and anterior saccule regions across the 7 TL groups, from 9 to 46 mm (n = 5–8 in each group). (**B**) The ratio of the HC number in the posterior saccule to that in the anterior saccule as a function of TL. (**C**) Regional divisions of the saccule. (**D**) HC densities were compared across TL groups using *post hoc* tests. A significant TL effect was revealed by one-way ANOVA (*P* < 0.001). (**E**) Comparison of TL-related HC density changes across different regions and TL groups. One-way ANOVA was performed for TL for each region, and a significant difference in HC density according to TL was observed in the four marginal regions (1, 6, 8 and 9; *P* *<* 0.001) but not in the central regions (2, 3, 4, 5 and 7; *P* > 0.05). *Post hoc* tests were performed to compare the HC of the 32–37 mm group for each region. *P < 0.05, **P < 0.01, ***P < 0.001.

To observe developmental changes, we labelled saccular HCs with Phalloidin and counted the number of HCs across 7 TL groups (TL = 9–10, 12–15, 23–26, 32–37, 39–40, or 42–46 mm, n = 5–8 in each group). In the youngest group (TL = 9–10 mm, corresponding to 30 dpf), each fish saccule had an average of 400 ± 26 (mean ± SEM) HCs (Fig. 3A). This number increased with age to a maximal value of 3288 ± 159 in the 39–40 mm TL group (14 months of age), representing an increase by approximately 8-fold in total, which corresponds to a net increase of 8 HCs/day. In older fish (TL ≥ 42 mm), the total HC number was 3023 ± 189 (Fig. 3A). However, the difference between the longest two TL groups was not statistically significant (Student’s *t* test, *t* = 0.9396, *P* = 0.3676).

To examine region-related differences in HC morphology, we divided the entire saccular epithelium into two portions (anterior and posterior) and 9 regions, as labelled in Fig. 3C. We found that the TL (age)-related change in HC number was similar between the two regions: it increased with TL until 39–40 mm and decreased thereafter (Fig. 3A). However, we observed a much greater increase in the HC number in the posterior saccule than in the anterior part. This result was further demonstrated by the change in the posterior/anterior HC number ratio with TL: from a ratio of 1.5 in the 9–15 mm TL group to a ratio of greater than 2 in the groups with a TL ≥ 23 mm (Fig. 3B). Consequently, more than two-thirds of the new HCs generated after hatching were observed in the posterior saccule. This finding indicates the importance of HCs in this region for the maturation of hearing in zebrafish.

To investigate developmental changes in HC density, we counted Phalloidin-labelled HCs in unit areas of 1,000 μm^2^ from 9 regions, as labelled in Fig. 3C. Five TL groups (n = 5–8 in each group) out of the seven used in the HC number study were chosen for HC density evaluation (Fig. 3D,E). We excluded the 9–10 mm TL group due to its small saccule size and the 39–40 mm TL group because of its similarity in HC density to the 32–37 mm TL group. TL (age)-related changes were expressed as the averaged HC density over 9 regions and were summarized in Fig. 3D. We identified an increase in the averaged density from the smallest TL group to the 32–37 mm TL group, followed by a decrease in the longest/oldest TL/age group. The change in the averaged density was analysed by a one-way ANOVA, which showed a clearly significant TL effect (*F*_4_,_16_ = 18.68, *P* < 0.001). The results of the *post hoc* pairwise comparisons are described in Fig. 3D. Figure 3E compares the HC densities across 9 different regions and the five TL groups. Clearly, the four marginal regions (1, 6, 8 and 9) displayed a higher density than the central regions (Fig. 3E). For each region, one-way ANOVA was performed for TL and the results showed significant difference (*P* < 0.001) for the marginal regions (regions 1, 6, 8 and 9) but not (*P* > 0.05) for the central regions (regions 2, 3, 4, 5 and 7). This finding was confirmed by Tukey’s *post hoc* tests. The asterisks in Fig. 3E indicate the significance level for each group relative to the value of the 32–37 mm group in each region. Based on these results, we suggest that the marginal regions are the major contributors to TL (age)-related changes in HC number and density.

**Figure 4:**
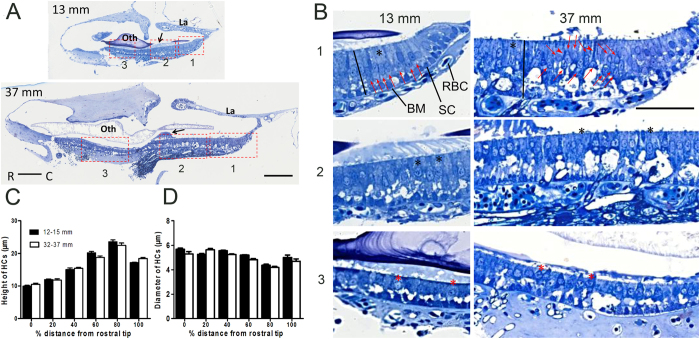
Comparison of HC morphology in semi-thin sections of the saccular epithelia of zebrafish with a TL of 13 mm or 37 mm. (**A**) Representative images of semi-thin sections. Scale bars: 100 μm; La: lagena; oth: otolith; black arrow: otolithic membrane. (**B**) Magnified images of different regions specified by the rectangles in A. In the 13 mm fish, the HCs were oriented in a monolayer across the entire saccule, whereas the HCs in the 37 mm fish were crowded, especially in the caudal region: the red arrows indicate HC nuclei located different distances from the surface (B, right upper panel), and the red arrowheads indicate HCs containing deeper nuclei that display cell bodies that are pushed above the nuclei. Scale bars: 50 μm; SC: supporting cells; BM: basement membrane; RBC: red blood cell; black line: the distance from the cuticular plate to the basement membrane; black asterisk: cylindrical HCs; red asterisk: pear-shaped HCs. (**C,D**) Differences in the location of HCs according to HC height and diameter in two TL groups. The HC height and diameter were measured at 6 locations across the saccule, from the rostral end (0% in distance) to the caudal end (100% in distance). At a point 80% of the distance from the rostral end (region 9 in Fig. 3C), the cell bodies of the HCs were the longest and had the smallest diameter. Two-way ANOVAs showed no significant TL effect for HC height (*P* = 0.58, n = 10 for each group) or diameter (*P* = 0.99, n = 10 for each group) but showed a significant effect of location for both HC height and diameter (*P* < 0.0001).

To further examine the density and the changes in the morphology of HCs in the saccule, semi-thin sections of the saccular epithelium along the rostral-caudal axis were collected from 12–15 mm and 32–37 mm TL fish (n = 3 for each TL group). Figure 4A shows representative images of such sections, and Fig. 4B shows magnified images of the selected regions labelled in Fig. 4A. Figure 4C,D demonstrate the comparisons of the height and diameter of HCs between the two TL groups of fish across 6 regions. Two-way ANOVAs were performed for the factors of region and either height or diameter. The results showed no TL effects (*P* = 0.58 for height and *P* = 0.99 for diameter), but they showed significant differences between regions according to both height (*F*_5, 54_ = 295.64, *P* < 0.0001, for region factor in the ANOVA for height and region) and diameter (*F*_5, 54_ = 46.63, *P* < 0.0001, for the region factor in the ANOVA for diameter and region). Similar to the reported findings in goldfish^31^, short, pear-shaped HCs were localized towards the rostral (anterior) end of the saccule (red asterisks in Fig. 4B), whereas taller cylindrical HCs were localized towards the caudal (posterior) end (black asterisks in Fig. 4B). Correspondingly, there was a significant increase in HC height from the rostral end to the caudal end. For example, the HC heights were shortest (9.98 ± 0.22 and 10.49 ± 0.38 μm for the two respective TL groups) at 0% distance from the rostral to the caudal end (region 1 in Fig. 3C) and were greatest (23.58 ± 0.55 and 22.47 ± 0.69 μm for the two respective TL groups) at 80% of the distance between the two ends (n = 10 for each group at each location, Fig. 4C). However, the diameter of HCs was smallest at the 80% distance (Fig. 4D).

Although no TL (age)-related effect was observed for either height or diameter, the structure of the saccular epithelium, especially the architecture of HC arrangement, appeared to change with TL (age) in addition to the changes in HC number. Figure 4B shows that in young fish with a TL of 13 mm, saccular HCs were lined up as a flat monolayer across the entire epithelium, whereas in adult fish with a TL of 37 mm, the HCs were more crowded, especially in the posterior (caudal) portion, where the nuclei were not the same distance from the surface (Fig. 4B: upper right panel, nuclei labelled with red arrows). These observations suggest an impact of increased HC density on the architecture of the epithelium. In fact, for those HCs with more deeply located nuclei, the cell bodies above the nuclei were “squeezed” into a smaller diameter than the cell bodies of HCs in the bottom portion (Fig. 4B: upper right panel, “squeezed” HCs are indicated by red arrowheads).

### Degeneration of HC bundles with ageing

**Figure 5:**
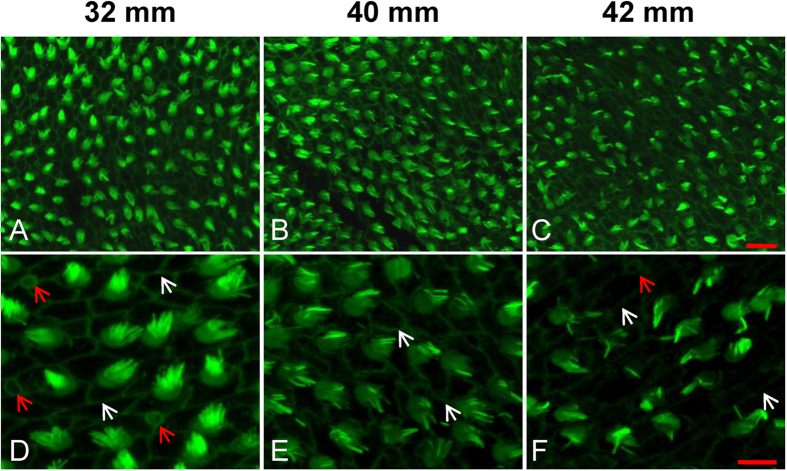
Representative images showing the degeneration of HC bundles with age. (**A**–**C**) Low magnification images of HC bundles in the anterior saccule of 32, 40 and 42 mm TL fish (6, 18 and 20 months, respectively). (**D**–**F**) High magnification images of (**A–C**). White arrows: cell-cell borders; red arrows: immature HCs; scale bars: 10 μm (**A**–**C**) or 5 μm (**D-F**).

The degeneration of HC bundles was observed in fish with a TL > 32 mm (32–37 mm, n = 8; 39–40 mm, n = 6; and 42–46 mm, n = 5), largely due to the loss of stereocilia. Figure 5 shows representative images of this change in the anterior saccule. The loss of HC bundles in the posterior and waist of the saccule is shown in Supplementary Fig. S5. The HC bundles of 32 mm TL fish (6 months of age; Fig. 5A,D) were clearly more abundant and more regularly oriented than those of older fish (TL 40 mm, 18 months; Fig. 5B–F). In the magnified image (Fig. 5E,F) of the older fish, the loss of HC stereocilia was observed (Fig. 5F). Further, clear Phalloidin-labelled cell-to-cell contact lines were observed in the 32 mm fish, which were blurred or indistinct in the 42 mm fish (white arrows in Fig. 5D–F). Moreover, many HCs in the 32 mm fish showed a typical immature epithelial morphology (characterized as a small surface outline of HCs and ciliary bundles that are distinctly shorter than the ciliary bundles of mature HCs). There were fewer HCs with this morphology in the older fish (red arrows in Fig. 5D,F), suggesting that the capability of adding HCs decreases with ageing. However, the changes in stereocilia were not statistically analysed due to the difficulty of quantifying this property. Supplementary Fig. S6 shows the entire saccule and HC bundles (including kinocilia and stereocilia).

### Ribeye b and ribbon expression in the saccule

**Figure 6:**
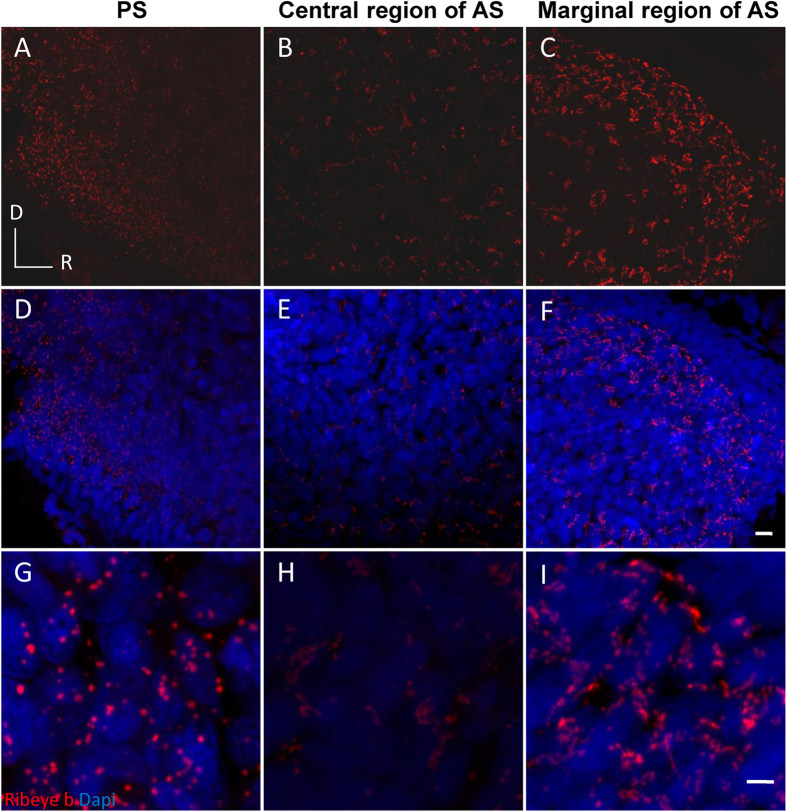
Representative images showing regional differences in Ribeye b expression in a 35 mm fish. **A** dispersed distribution of round puncta (red) were observed in the posterior saccule (**A,D,G**). Clustered spots of labelling were observed in the marginal anterior saccule (**C,F,I**). Smaller but still clustered puncta were observed in the central region of the anterior saccule (**B,E,H**). (**G–I**) are magnified from (**D–F**), respectively. AS: anterior saccule; PS: posterior saccule; R and D: rostral and dorsal, respectively; scale bars: 5 μm (**A–F**) or 2 μm (**G–I**).

**Figure 7:**
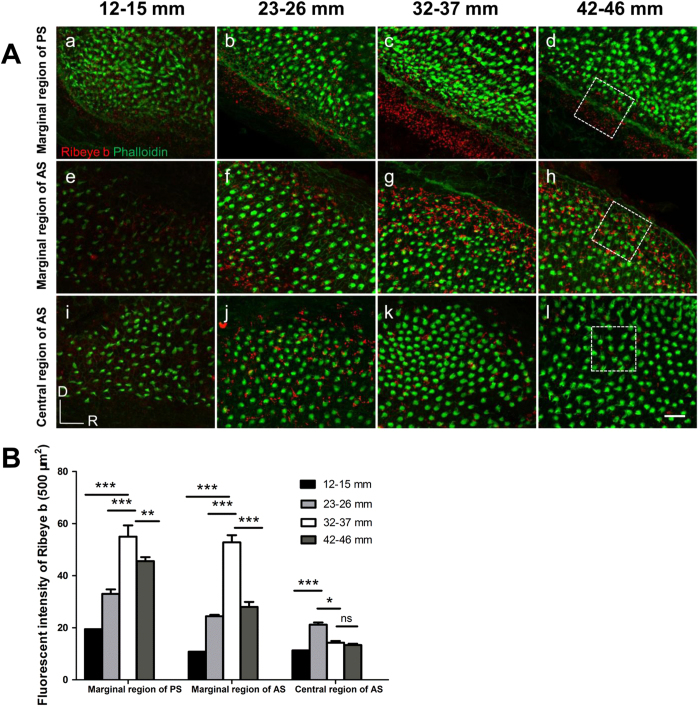
Representative images of Ribeye b staining in distinct regions of different TL groups. (**A**) The ventral marginal region of the posterior saccule (**a**–**d**), the dorsal marginal region of the anterior saccule (**e**–**h**), and the central region of the anterior saccule (**i**–**l**), corresponding to regions 8, 1 and 3, respectively, as labelled in Fig. 3C. White dotted rectangles: the areas used for fluorescence intensity analysis, which is presented in B. (**B**) TL (age)-related changes in the fluorescence intensity of Ribeye b protein immunolabelling in three regions. An increase in the fluorescence intensity of Ribeye b immunolabelling was visible in the two marginal regions (regions 1 and 8) from the 12–15 mm TL group to the 32–37 mm TL group (**a–c** and **e–g**), followed by a decrease in the 42–46 mm TL group that was accompanied by a decrease in HC density (**d,h**). In the central region (region 3), a decrease in fluorescence intensity was observed in the 32–37 mm TL group (k compared to j). The asterisks indicate the significance of the differences within each region across different TL groups based on ANOVA followed by *post hoc* tests. *P < 0.05, **P < 0.01, ***P < 0.001.

There are two ribeye genes expressed in the synaptic ribbons of zebrafish sensory cells: *ribeye a* and *b*. Ribeye b is expressed most strongly in the inner ear^32^. Immunostaining for this protein in the posterior saccule (Fig. 6A,D,G) showed what appeared to be rounded puncta that were distinguishable from each other as individual ribbons. However, immunostaining for Ribeye b appeared as clustered, irregular puncta in the anterior saccule that were larger in the marginal region (Fig. 6C,F,I) and smaller in the central region (Fig. 6B,E,H). In those areas, it was difficult to differentiate puncta corresponding to distinct ribbons. As shown in Figs 6 and 7A and Supplementary Fig. S7, this expression pattern of Ribeye b in the saccule did not change as the fish developed.

To quantify the TL (age)-related changes in Ribeye b expression, we compared the average fluorescence intensity of this protein in a fixed area of the saccular epithelium across the 4 TL groups (TL = 12–15, 22–26, 32–37 or 42–46 mm, n = 5 for each group, Fig. 7) and the 3 regions (posterior, anterior central and anterior marginal) using a two-way ANOVA for the factors TL and region. The effects of both factors (TL: *F*_3, 36_ = 101.04, *P* < 0.0001; region: *F*_2, 36_ = 329.25, *P* < 0.0001) and the interaction between the two factors (*F*_6, 36_ = 26.89, *P* < 0.0001) were significant. Tukey’s *post hoc* tests revealed region-specific differences between the TL groups (Fig. 7B). In the two marginal regions (1, 8), the fluorescence signal of Ribeye b immunostaining increased from a TL of 12–15 mm to a TL of 32–37 mm and then decreased at a TL of 42 mm, this pattern roughly coincided with the TL (age)-related change in HC number, as shown in Fig. 3A. In contrast, in the central region of the anterior saccule, the overall fluorescence signal was weaker in the younger fish (in the 32–37 mm group). This result suggests that there is a difference in Ribeye b expression in this region according to size.

**Figure 8:**
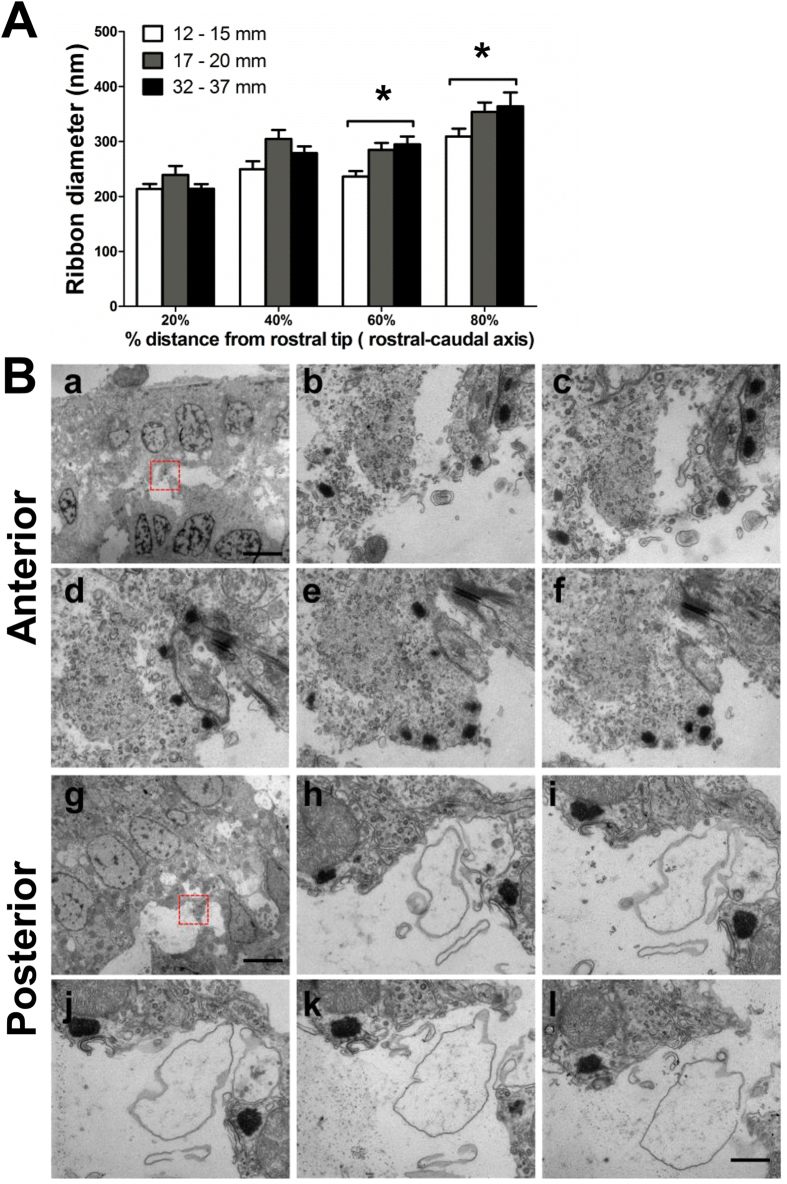
Ribbon diameter across different TLs and regions. (**A**) Comparison across three TL groups at four distances from the rostral end (0%, 20%, 60% and 80% distances) of the sensory epithelium (ribbon number observed at the four distances: n = 52, 36, 33 and 22, respectively, for 12–15 mm TL fish; n = 22, 24, 39 and 30, respectively, for 17–20 mm TL fish; and n = 52, 30, 37 and 16, respectively, for 32–37 mm TL fish). *P < 0.05, *post hoc* tests within each distance between the indicated TL groups. (**B**) Representative TEM images of serial sections. The HCs of the anterior saccule contained small ribbons that were close to each other (**a–f**), in contrast to the large, rounded and dispersed ribbons observed in HCs in the posterior saccule (**g–l**). The red rectangles in (**a,g**) indicate the regions that were magnified and observed in the serial sections (**b–f** and **h–l**, respectively). Scale bars: 5 μm (a and g); 500 nm in all magnified images.

To validate the observed changes in the pre-synaptic ribbon, transmission electron microscopy (TEM) was used to observe ribbon morphology. The ribbon diameters of the saccular HCs were examined across 4 locations (20%, 40%, 60%, and 80% of the distance along the rostral-to-caudal axis of the saccule epithelium) and 3 TL groups (TL = 12–15, 17–20 and 32–37 mm). For each region in each TL group, the ribbons were measured from multiple HCs that were collected from at least 3 fish. Similar to immunohistological observation, this experiment revealed that the ribbon diameter appeared to increase along the rostral-caudal axis (Fig. 8A): HCs in the anterior saccule contained smaller, clustered ribbons (Fig. 8B, a–f), whereas those in the posterior saccule contained larger, round, dispersed ribbons (Fig. 8B). This trend manifested as a significant main effect of distance (region) (*F*_3, 383_ = 33.29, *P* < 0.0001) based on two-way ANOVA, which also demonstrated an effect of TL (*F*_2, 383_ = 11.33, *P* < 0.0001). *Post hoc* pairwise comparisons showed significant differences in ribbon diameters between the 12–15 and 32–37 mm TL groups at both the 60% and 80% distances (both *P* < 0.05, as indicated by asterisks in Fig. 8A).

## Discussion

In the present study, we examined the hearing ability changes in zebrafish with a TL ranging from 12 to 46 mm (corresponding to an age from 40 dpf to 20 months post-fertilization) and assessed the age-related morphological variations in saccular HCs. The major findings are summarized as follows: 1, the hearing sensitivity, but not the hearing frequency range, of zebrafish changes with TL (or age) (Fig. 2); 2, the HC number continues to increase until late in adulthood, and more than two-thirds of new HCs are located in the posterior saccule (Fig. 3A,B); 3, there are evident region-specific differences in HC height and diameter (Fig. 4C,D) and TL (age)-related differences in HC density (Fig. 3D,E), ribbon protein expression pattern (Figs 6 and 7) and ribbon size (Fig. 8); 4, the TL (age)-related changes in HC number and density and Ribeye b protein expression are consistent with the AEP threshold change; and 5, the subsequent elevation in the AEP thresholds among the longest TL group are consistent with their loss of hair bundles (Fig. 5).

### Hearing sensitivity change

Ontogenetic changes in hearing sensitivity have been examined behaviourally and/or eletrophysiologically in a few fish species. However, the overall outcomes of these previous reports present a complicated and conflicted picture^10^,^24^,^26^,^33^,^34^,^35^,^36^,^37^, as some showed no change in hearing sensitivity^11^,^33^, but many others suggested a size/age-related increase^24^,^26^,^34^,^35^,^36^, or a slight decrease in hearing sensitivity with increasing size^37^. The comparison in this report focuses on fish that belong to the group of “hearing specialists”, in which Weberian ossicles develop to form a connection between the swim bladder and the hearing sensory organ. It is believed that the connection between the ossicles connection and the swim bladder enhances hearing sensitivity and broadens the hearing frequency range in this group of fish^9^. Therefore, several reports have attempted to determine the relationship between hearing and the Weberian ossicles during individual development^11^,^26^,^38^,^39^.

In zebrafish, only two studies, by Higgs *et al.*, used AEP recordings to observe the changes in hearing ability that occur with size/age^10^,^11^. No changes in hearing response threshold, latency, or amplitude were found in zebrafish with a TL from 10 to 35 mm. However, the best hearing frequency ranges reported in their studies are similar to those reported in our present study. This consistency indicates that electromagnetic contamination (as indicated by noisy AEP waveforms) might have masked the potential changes in noise-sensitive AEP thresholds.

In addition to the AEP technique, various methods have been used to evaluate hearing abilities in zebrafish^12^,^14^,^15^. Zeddies and Fay found no age-related changes in startle response thresholds or the hearing frequency range (100–1,200 Hz) in zebrafish from 5 dpf to adulthood (8 months of age, 31 mm in TL)^12^. It is questionable whether the startle response threshold represents hearing sensitivity because the startle response is generally considered as a supra-threshold phenomenon that shows a much higher thresholds, at least in mammals, than the thresholds obtained from electrophysiological recordings^40^,^41^. To the best of our knowledge, no report to date has compared the hearing threshold for a startle response with the hearing threshold for an AEP in any fish species. However, the hearing thresholds reported in the study by Zeddies and Fay that used a startle response were between 145 and 180 dBw across frequencies; this range much higher than that which is reported in the present study (between 105 and 140 dBw).

Age/size-related hearing threshold changes similar to those found in the present study have been observed in other hearing specialist fish species using the AEP technique. These species include the labyrinth fish *Trichopsis vittata*^42^, the squeaker catfish *Synodontis schoutedeni*^24^ and, more recently, the catfish *Lophiobagrus cyclurus*^26^. However, contradictory reports also exist. For example, a previous study of goldfish showed no changes in hearing sensitivity between older juvenile (45–48 mm standard length, SL) and adult goldfish (110–120 mm SL) based on a shock-conditioning technique^33^. It is unclear whether these negative results were due to the technique or to the limited sample size used in this study.

### Frequency range change

The available data do not present a clear picture regarding the age/size-related changes in the hearing frequency range. In the present study, we did not detect a significant change in the hearing frequency range between juvenile and adult fish. This result is in agreement with the observations mentioned above by Zeddies and Fay, who studied startle response^12^, and with the findings of a study of the squeaker catfish *S.schoutedeni*^24^. However, an expansion in the hearing frequency range with age/size was reported in one study of zebrafish^11^ and in one study of a species of catfish *L.cyclurus*^26^. Further investigation is needed to determine the reasons for these discrepancies across different studies within and between fish species. Additionally, AEPs were recordable up to a much higher frequency limit (12 kHz) than previously reported, an upper threshold of approximately 4 kHz in zebrafish^10^,^11^ and closely related goldfish^25^. The reasons for this difference are not entirely clear, but there are likely multiple reasons, including the use of different speakers (the AQ399 speaker that we used provids a higher sound level at the high frequency end compared to the UH30 speaker, which was used by Higgs) and the differences in animal treatments (the fish were pinned and mildly anesthetized in our experiments). Further information is provided in the supplemental materials (Supplementary Fig. S8 and Table S2).

### The role of Weberian ossicles

It has been widely accepted that the Weberian ossicles and the swim bladder are responsible for the high sound sensitivity of fish species containing this apparatus relative to other fish species^9^. Because these structures develop with age/size, hearing thresholds should decrease as the animal matures to adulthood. It was reported that the Weberian ossicles are first evident in zebrafish with a TL of 7 mm and that they are mature in fish with a TL of 19.5 mm^11^. Therefore, the decreased AEP threshold observed in our sample up to a TL of 32 mm can be partially, although not fully, explained by the development of the Weberian ossicles and swim bladder.

The effect of the Weberian ossicles and the swim bladder on the hearing frequency range of zebrafish is debatable. In a study by Higgs^11^, an expansion in the hearing frequency range was reported to be related to the development of the Weberian ossicles: the frequency range was expanded from only 200 Hz in 10–13 mm TL fish to up to 4 kHz in fish with a TL greater than 20 mm. Similarly, in a species of catfish, *L.cyclurus*, the expansion of the hearing frequency range with changing hearing thresholds was identified^26^. The smallest fish observed in this study lacked a fully developed chain of Weberian ossicles and exhibited a hearing frequency range of up to 2 kHz, whereas the hearing frequency range of older fish was expanded up to 6 kHz. In contrast, Zeddies and Fay observed that zebrafish showed no age-related changes in the hearing frequency range^12^, and identical results were obtained from a study of the squeaker catfish *S. schoutedeni*^24^, in which all subjects had fully developed Weberian ossicles. Our study included zebrafish with a TL from 12 to 46 mm; these fish had Weberian ossicles ranging from immature to mature stages. We did not observe an expansion in the hearing frequency range with TL during the development of the Weberian ossicles. This finding suggests that the hearing frequency range of zebrafish may not be fully dependent on the ossicles. Alternative mechanisms of sound conduction, as described by Ladich^38^, are likely to contribute to the hearing frequency range in hearing specialists. Further research is needed to clarify this issue.

### The roles of other structures

Other age/size-related changes observed in this study included changes in HC number and density and in the size of the sensory epithelium (Figs 3 and 4A), corresponding changes in the expression level of the ribbon protein Ribeye b (Figs 6 and 7) and the changes in stereocilia (Fig. 5).

Fish, different from most other vertebrates, continue to accumulate HCs in their inner ear after hatching^4^,^6^,^43^,^44^,^45^,^46^. However, the functional role of those newly added HCs and how changes in HC number are related to changes in hearing sensitivity and the potential hearing frequency range are unclear. A few studies have examined the relationship between HC number in the fish inner ear and hearing sensitivity^10^,^14^,^46^,^47^. Recently, a study on zebrafish aged 3–7 dpf demonstrated improved hearing sensitivity on tests using the microphonic potential method; this improvement correlated with an increased number and density of saccular HCs^14^. Coffin *et al.*^46^ found that in the plainfin midshipman fish *Porichthys notatus*, saccular-specific HC additions were concurrent with the seasonal changes in reproductive female auditory sensitivity.

Based on our data, the AEP threshold continually decreased with TL up to 32–37 mm (Fig. 2B); this pattern is roughly consistent with the increase in HC number that was observed up to a TL of 39–40 mm (Fig. 3A). The present study demonstrated a similar trend in the changes in Ribeye b expression according to TL (Fig. 7B). Moreover, the hearing loss observed in the older fish was consistent with the observed decreases in HC number, Ribeye b expression and stereocilia abundance. Taken together, the data reported to date support the concept that HC accumulation and, possibly, changes in ribbon synapses contribute to the age/size-related improvements in hearing sensitivity. Further investigation is needed to determine how these structures contribute to the sensitivity of fish hearing. In particular, the hearing frequency range of zebrafish is a very difficult issue that needs to be addressed because it is not entirely clear whether or how the hearing frequency range might change with age/size.

We used AEPs to study zebrafish hearing during development and analysed both hearing sensitivity and hearing frequency range over a large TL range. The overall pattern of the hearing sensitivity of zebrafish observed in this study present a pattern similar to that of the squeaker catfish *S. schoutedeni*. In addition, our data suggest that in addition to the role of Weberian ossicles, changes in HC number, Ribeye b protein expression, and stereocilia abundance each contribute to hearing sensitivity changes over time.

## Methods

### A statement identifying experimental approval by an institutional and/or licensing committee

All animal experiments were performed according to the guidelines and the approval of the Committee of Experimental Animal Service, Shanghai, China.

### Experimental animals

AB line zebrafish were bred and reared in filtered aquaria at 28 ± 1 °C in our fish colony. The fish were maintained under a 12:12 light:dark cycle and were fed three times per day.

### Auditory sensitivity measurements

The AEP recordings were conducted in a rectangular tank (100 cm × 42 cm × 50 cm) that contained two chambers, including a small chamber housing a grounded speaker that was isolated by magnetic shielding. The border between the two chambers was made acoustically transparent by mesh holes that were present in the area facing the speaker diaphragm. The holes were covered with a thin plastic membrane to physically separate the water between the two chambers. The tank was placed in a soundproof chamber (see Supplementary Fig. S8).

Zebrafish were temporarily anesthetized and immobilized in 0.01% tricaine methanesulphonate (MS-222; Sigma-Aldrich, St. Louis, MO, USA). Then, they were mounted and pinned on a 10 cm dish padded with silica gel. The dish containing the fish was fixed on a plastic strip 5 cm below the surface of the water and 25 cm away from the underwater speaker. The temperature of the water was 25 ± 1 °C. Three electrodes (tungsten electrode, diameter 0.005 in.; resistance, 5 mega-Ohms; A-M Systems Inc., WA, USA; see Supplementary Table S2) were used for recording. The electrodes were positioned under a surgical microscope using a micromanipulator. The recording electrode was inserted into the dorsal surface of the fish, just behind the brainstem; the reference electrode was inserted into the muscles of the dorsal fin; and the grounding electrode was inserted near the tail. Both sound presentation and AEP recordings were accomplished using Tucker-Davis Technologies hardware and software (TDT, Alachua, FL, USA). Stimuli were evoked from a PC to the underwater speaker (AQ339; Clark Synthesis, CA, USA) and consisted of tone bursts at 100, 200, 400, 600, 800, 1,000, 2,000, 3,000, 4,000, 6,000, 8,000, or 12,000 Hz. The tone bursts had a 2 ms rise/fall time, were 20 ms in duration and were gated through a Blackman window. A hydrophone (TC 4032; frequency range, 10 Hz to 80 kHz ± 2.5 dBw; receiving sensitivity, −170 dBw; Reson Inc., Slangerup, Denmark) was used for the calibration of the sound pressure level (SPL). To this end, the microphone was placed at the position that would be occupied by the fish in the recording tank. Tone bursts of different frequencies were initially presented by driving the acoustic amplifier with 1 Vrms electrical signals. The exact SPLs were calculated based on the microphone output and sensitivity using a reference of 1 μPa. A table of correction values was generated for the frequencies tested by calculating the differences between the SPLs produced by 1Vrms and the highest sound level (i.e., 160 dBw). Then, the correction values were used to set up an attenuator to control the sound levels during the AEP test.

The acquired AEP signals were fed into a pre-amplifier (RA16PA, TDT), in which the signals were amplified by 20-fold, filtered between 0.1 and 1 kHz, and digitized. The output of this pre-amplifier was sent to a real-time processor (RA16BA, TDT). The signal processing by all TDT hardware used for sound delivery (RP2.1) and AEP acquisition (RA16PA and BA) was controlled by a PC using BioSigRP software. At each frequency, the AEP was recorded in a descending sequence of SPLs in 5 dB steps from 160 dBw to the hearing response threshold, which was defined as the lowest level at which a visible and repeatable AEP wave was observed in two averaged trials. In each trial, the responses were averaged over 1,000 sweeps.

### Fluorescence staining

Briefly, fish heads were fixed with 4% paraformaldehyde at 4 °C overnight. After rinsing the tissue three times with 0.25% PBST, the sensory epithelia of the saccules were dissected according to a previous report^48^. For HC counting, the epithelia of the saccules were incubated for 15 minutes in Alexa Fluor 488 Phalloidin (Invitrogen) at 4 °C. For Ribeye b immunofluorescence labelling, the epithelia of the saccules were permeabilized using 1% Triton X-100 at room temperature for 2 hours. Then, the samples were blocked with 10% goat serum in PBS for 1 hour, followed by incubation in the primary antibody (mouse anti-zebrafish Ribeye b monoclonal antibody, kindly provided by Dr. T. Nicolson, Oregon Health & Science University) at 4 °C for 18 hours. After the tissue was rinsed with 0.25% PBST for 2 hours, the samples were stained with a secondary antibody coupled to DyLight 549 (Jackson ImmunoResearch) for 2 hours at room temperature. Finally, the samples were stained with DAPI (Invitrogen).

### Confocal microscopy and quantification

Confocal images were acquired using a Zeiss LSM 710 microscope (Carl Zeiss Microimaging, Jane, Germany) using a 20 × or 100 × oil immersion objective lens. DAPI (405 nm), Alexa 488 (488 nm) or DyLight 549 was visualized via blue-violet diode, Argon-ion and Green HeNe excitation, respectively. Z-stack images (0.3 μm apart over 5–10 μm) were transformed into TIFF images, and the mean fluorescence intensity of Ribeye b immunolabelling was determined using ZEN 2011 software (Carl Zeiss) with background correction. Three 500 μm^2^ regions of interest were manually selected, as marked by white dotted rectangles in (Fig. 7A,d,h,l). The mean optical density values in each region were calculated.

### TEM and semi-thin section processing

TEM of ribbon synapses was performed according to routine procedures^49^. Semi-thin sections (1 μm) were generated using a Leica UC7 Ultramicrotome (Leica) and stained with 1% toluidine blue in 1% sodium borate buffer. Digital images were acquired using a Zeiss LSM 710 microscope, and the height and diameter of HCs were analysed using ZEN 2011 software (Carl Zeiss).

### Statistical analysis

The data were analysed using GraphPad Prism (GraphPad Software Inc.) and SPSS software (SPSS Inc.). Mean hearing thresholds were determined for each group and at each frequency, and audiograms and histograms were drawn using GraphPad Prism 5. We used ANOVA to determine whether the average HC density varied significantly according to TL or region. Tukey’s *post hoc* test was used for pairwise comparisons between TL groups or regions when significant main effects were found. Two-way ANOVAs were used to evaluate HC height and diameter, fluorescence intensity of Ribeye b immunolabelling, and ribbon diameter considering the factors TL and region, followed by Tukey’s *post hoc* tests when significant main effects were found.

## Additional Information

**How to cite this article**: Wang, J. *et al.* Ontogenetic development of the auditory sensory organ in zebrafish (*Danio rerio*): changes in hearing sensitivity and related morphology. *Sci. Rep.* **5**, 15943; doi: 10.1038/srep15943 (2015).

## Supplementary Material

Supplementary Information

## Figures and Tables

**Table 1:** Zebrafish total length and corresponding age.

Total length (TL)	Approximate age
9–10 mm	30–35 dpf
12–15 mm	40–50 dpf
17–20 mm	50–60 dpf
22–26 mm	2–3 months post-fertilization
32–37 mm	6–10 months post-fertilization
39–40 mm	12–14 months post-fertilization
42–46 mm	16–20 months post-fertilization

dpf: days post-fertilization.
