# Supplementary material for: Ontogenetic development of the auditory sensory organ in zebrafish (Danio rerio): changes in hearing sensitivity and related morphology
Source: Sci Rep. 2015 Nov 3;5:15943. doi: 10.1038/srep15943 (PMC4630651; doi:10.1038/srep15943)
Supplement: Supplementary Information [file srep15943-s1.doc]

**Ontogenetic development of the auditory sensory organ in zebrafish (*Danio rerio*): changes in hearing sensitivity and related morphology**

Jiping Wang^1,†^, Qiang Song^1,†^, Dongzhen Yu^1^, Guang Yang^1^, Li Xia^1^, Kaiming Su^1^, Haibo Shi^1, *^, Jian Wang^1,2,*^ and Shankai Yin^1, *^

1. Department of Otolaryngology, Affiliated Sixth People's Hospital of Shanghai Jiao Tong University, Otolaryngology Institute of Shanghai Jiao Tong University, Shanghai 200233, China.

2. School of Human Communication Disorder, Dalhousie University, Halifax, Nova Scotia, Canada.

^†^ These authors contributed equally to the present study.

^*^Authors for correspondence

**Shankai Yin**

Department of Otolaryngology, Affiliated Sixth People's Hospital of Shanghai Jiao Tong University, Otolaryngology Institute of Shanghai Jiao Tong University, Shanghai 200233, China.

Tel: +862164834143.

Fax: +862164834143.

Email: yinshankai@china.com

**Jian Wang**

School of Human Communication Disorder, Dalhousie University, Halifax, Nova Scotia, Canada.

Tel: +19024945149.

Fax: +19024945151.

Email: Jian.Wang@Dal.Ca

**Haibo Shi**

Department of Otolaryngology, Affiliated Sixth People's Hospital of Shanghai Jiao Tong University, Otolaryngology Institute of Shanghai Jiao Tong University, Shanghai 200233, China.

Tel: +862164834143.

Fax: +862164834143.

Email: haibo99@hotmail.com

**
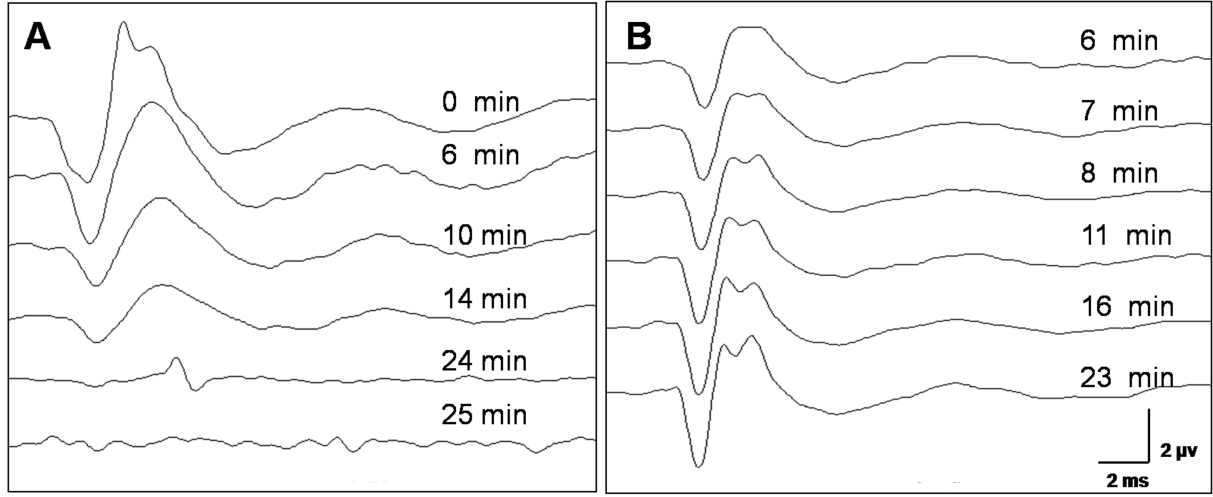
**

**Supplementary Fig. S1**│ **AEP verification under deep anaesthesia.** (A) Serial recordings obtained from a fish at different time points after anaesthesia with high dose (0.1%) tricaine methanesulphonate (MS-222; Sigma-Aldrich, St. Louis, MO, USA). The AEP was initially detectable and then disappeared (4,000 Hz 160 dBw, the highest AEP intensity used in this study). (B) Serial recordings from a fish that was weaned off of the deep anaesthesia as performed in (A). The AEP amplitude increased and the latency decreased with a time delay after removal of anaesthesia. The test used in (A) was performed on 6 fish, and the test used in (B) was performed on 4 fish (the other two tested fish died). The frequency of tone bursts in (A) was 4 kHz, and that in (B) was 7 kHz. The impact of anaesthesia differentiated AEPs from artefacts.

**
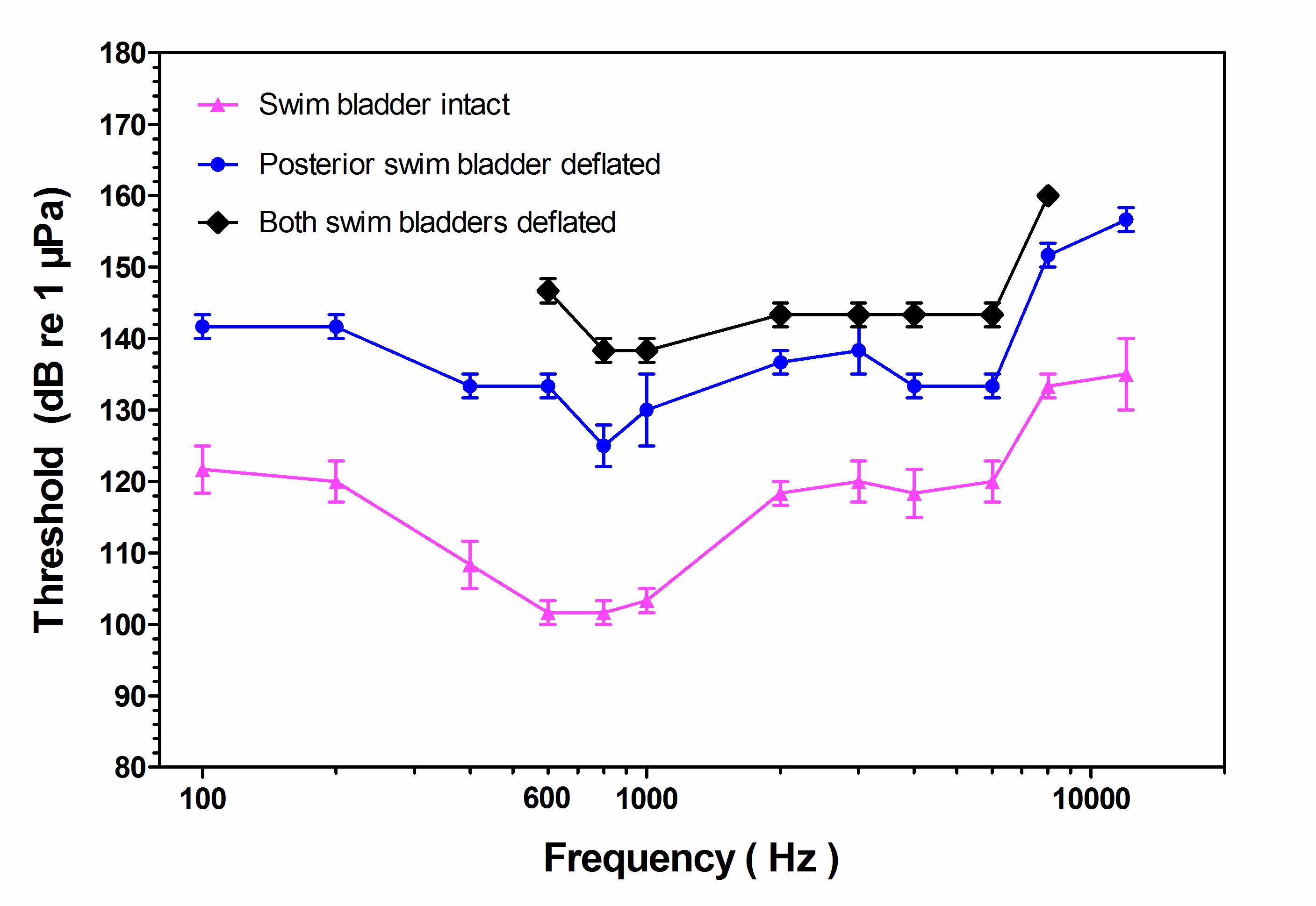
**

**Supplementary Fig. S2**│ **AEP verification by deflating the swim bladders.** The tests were repeated in 4 fish with a TL of 31-40 mm. The bladders were penetrated with an ultra-fine needle. Representative results show that deflation largely elevated the AEP threshold, especially when both bladders were deflated. This impact of swim bladder deflation on the AEP thresholds suggests a biological, rather than an artefactual, origin of the AEPs.

**
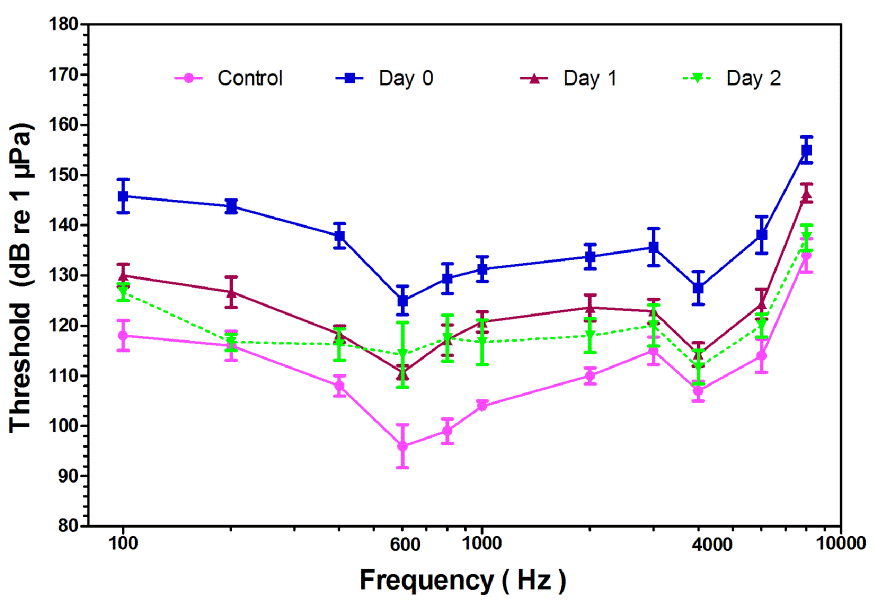
**

**Supplementary Fig. S3**│ **Verification of the AEP results by evaluating the hearing threshold changes induced by acoustic overstimulation.** Fish (TL = 32-42 mm, n = 5-8 in each group) were tested 0, 1 and 2 days after exposure and were compared with controls. The traumatizing tone, which was 150 Hz at 160 dBw for 60 hours, was delivered via an underwater speaker (LL9816, Lubell Labs Inc., OH, USA). Threshold shifts were observed primarily on 0 day after noise and were partially recovered 2 days later. These results confirmed the biological, rather than artefactual, nature of the responses represented by the recorded waveforms.

**
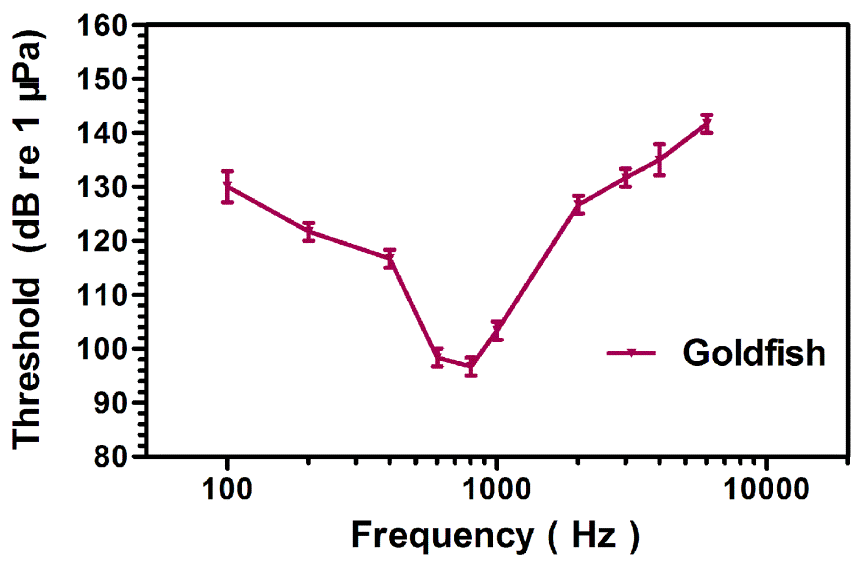
**

**Supplementary Fig. S4** │**Verification of AEP detection in goldfish.** Goldfish (TL 38-54 mm, n = 8) were tested as a reference, and the results demonstrated threshold audiograms and best hearing frequency ranges that were similar to those reported in previous studies.

**
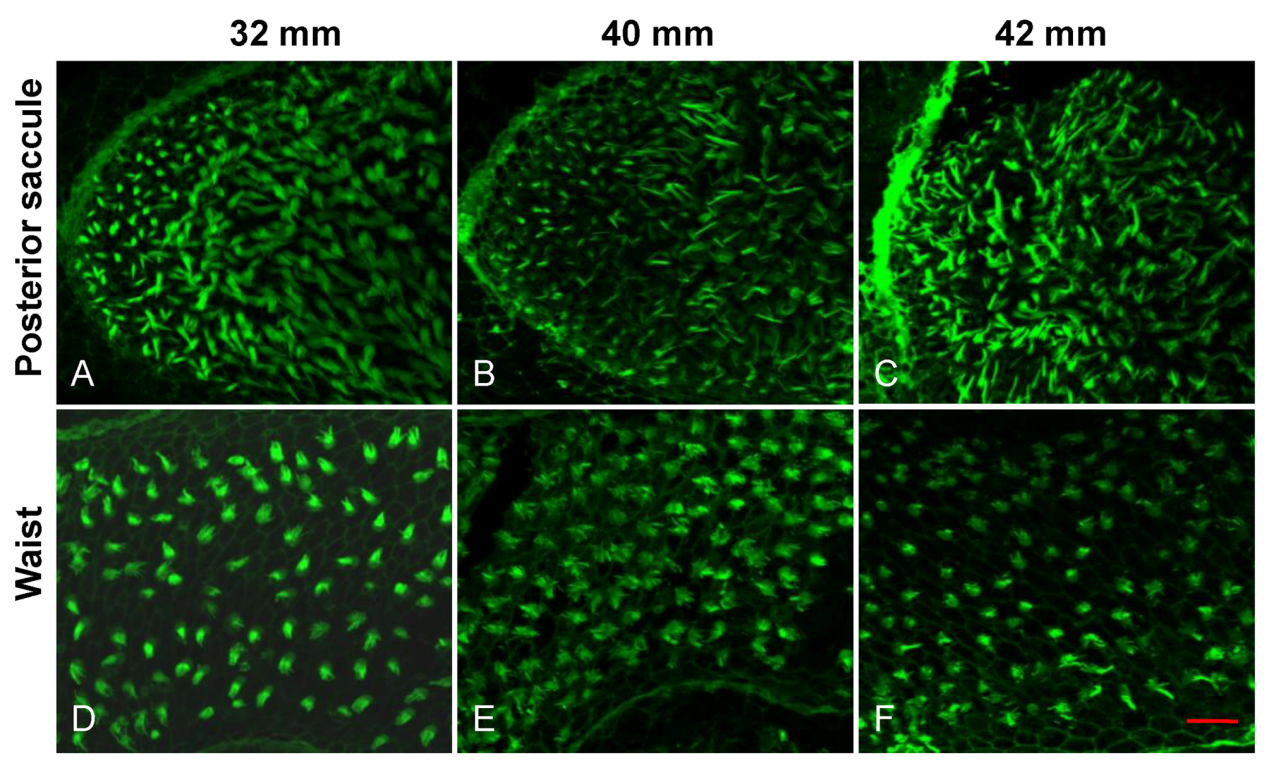
**

**Supplementary Fig. S5**│ **Images of HC bundles in the saccule waist and the posterior saccule (supplemental to Fig. 5).** Scale bars: 10 μm (A-F).

**
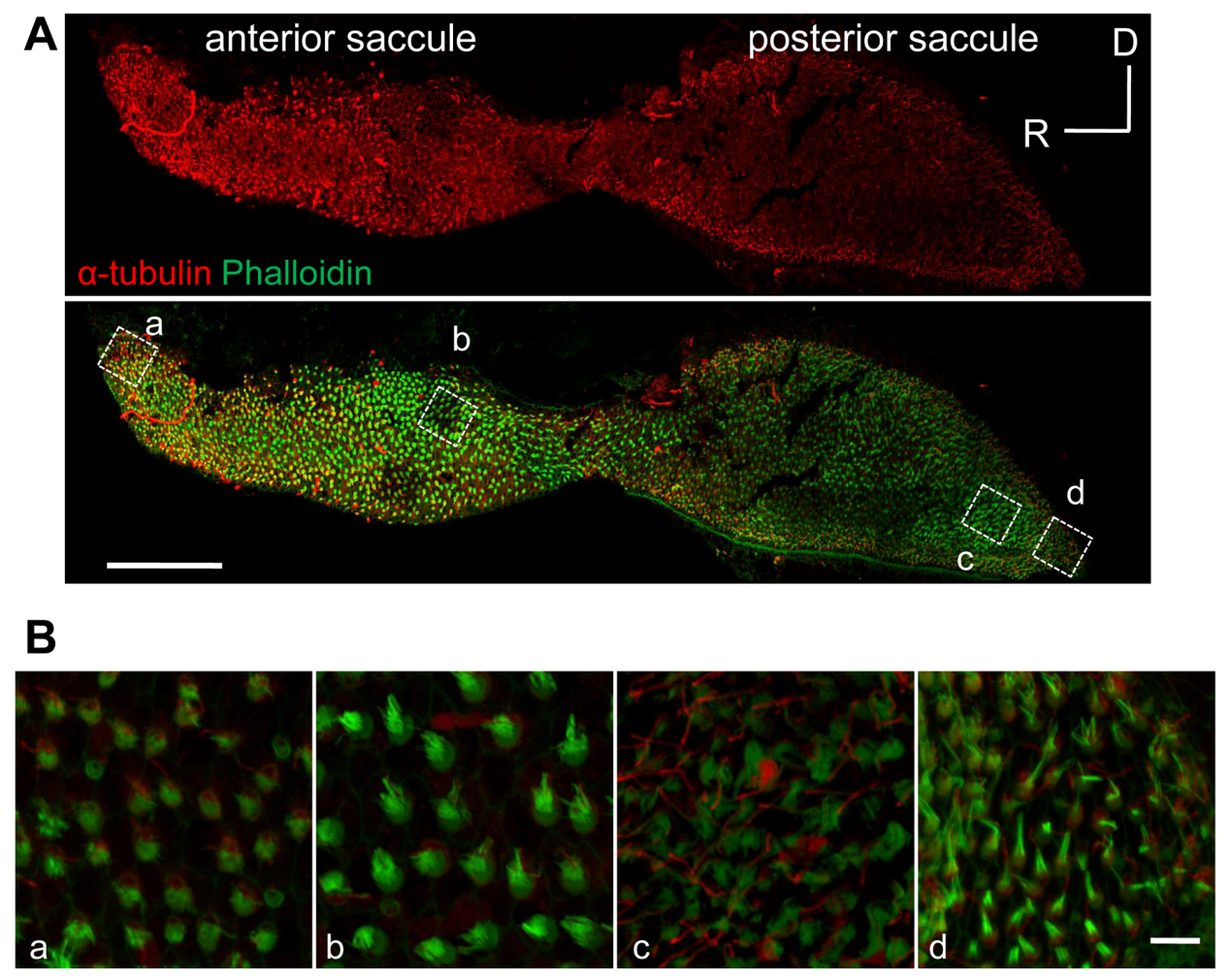
**

**Supplementary Fig. S6**│ **Images of saccular HC bundles.** (A) Representative images of kinocilia, which were immunostained for α-tubulin (red), and stereocilia, which were counterstained with phalloidin (green). Scale bars: 100 μm. Fish TL = 41 mm. (B) Magnified images from regions inside the dotted rectangles in the lower panel of (A). Scale bars: 5 μm (a-d).

**
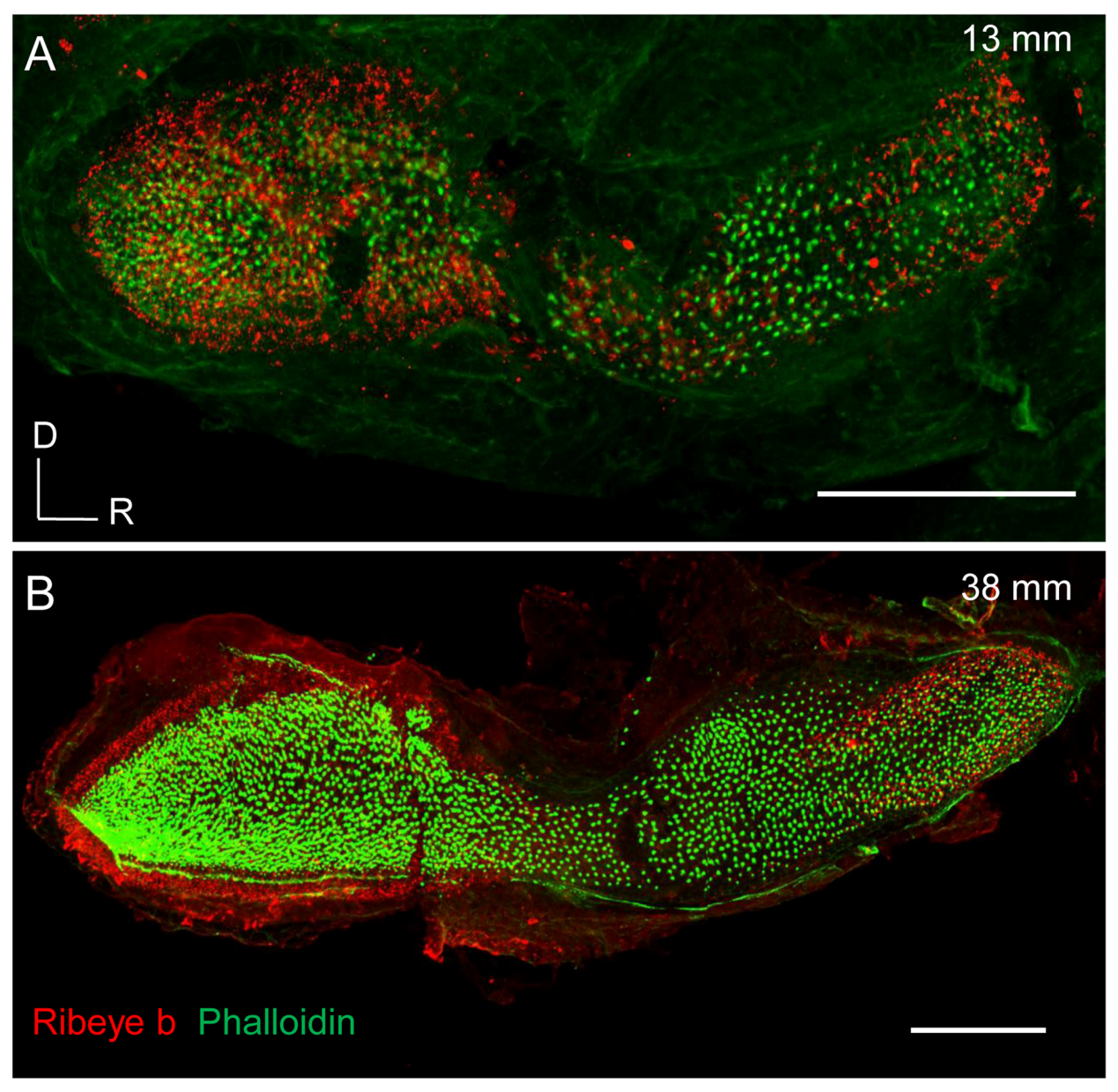
**

**Supplementary Fig. S7**│ **Immunofluorescence labelling for Ribeye b.** Representative images of saccules from young (A) and old zebrafish (B) showing Ribeye b expression (red) and hair bundles stained with phalloidin (green). Scale bars: 100 μm.


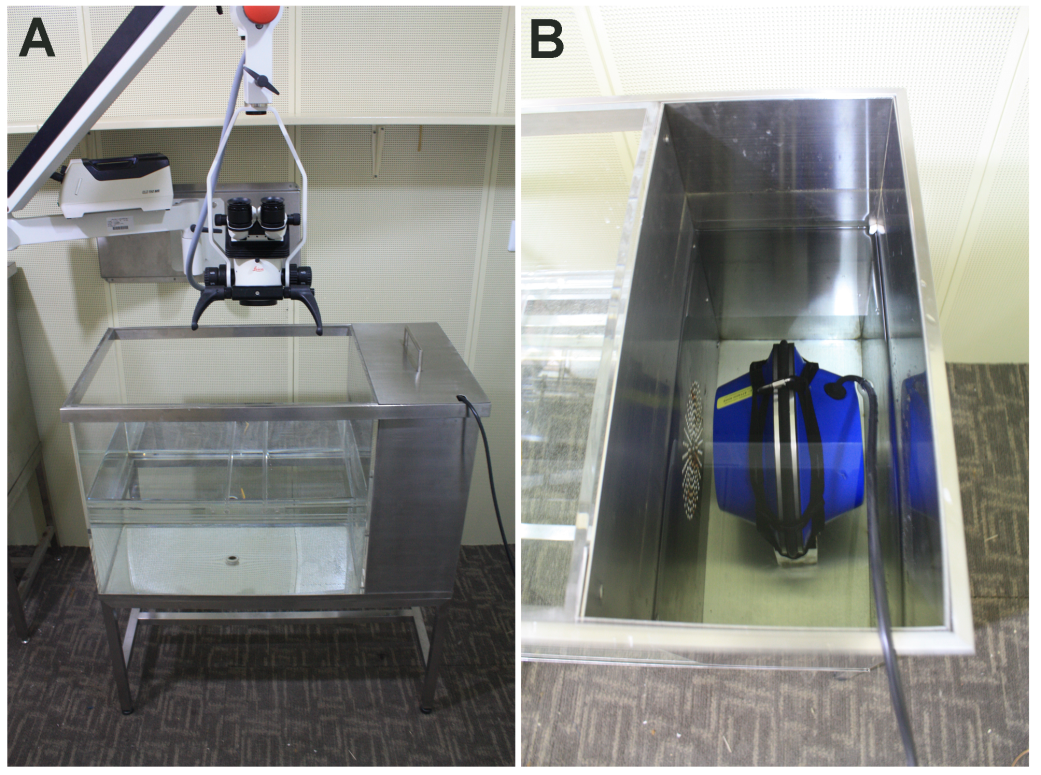


**Supplementary Fig. S8**│ **The AEP testing apparatus.** (A) Above view of the AEP testing tank and surgical microscope. (B) Open view of the tank housing the speaker. The wall of this tank is composed of steel plates for electromagnetic shielding.

**
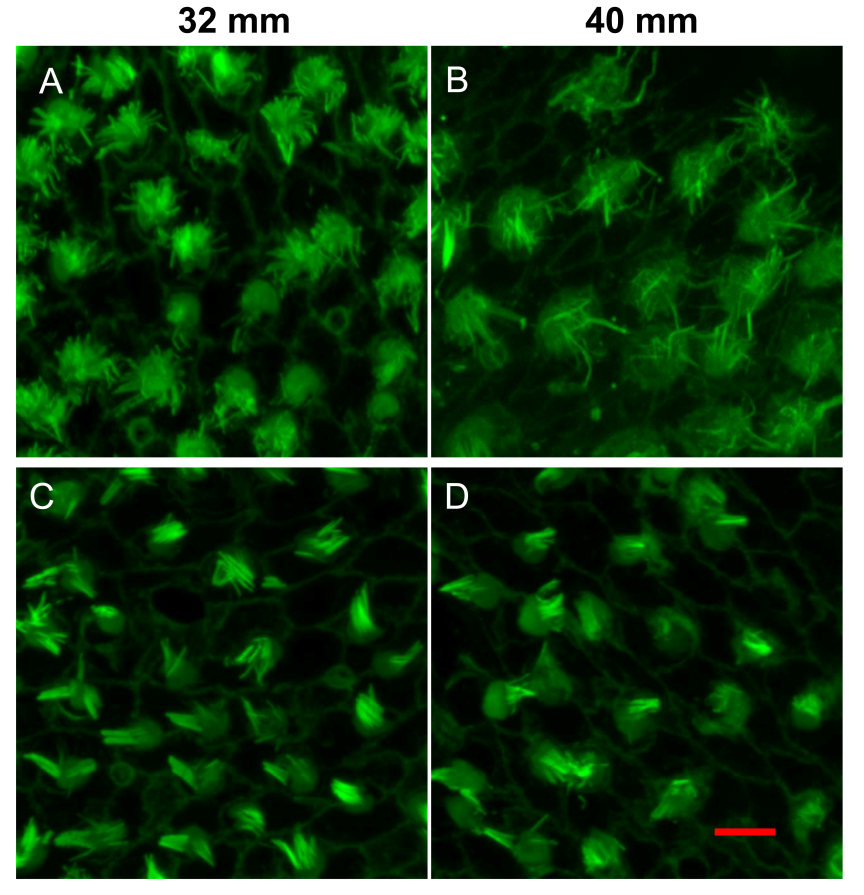
**

**Supplementary Fig. S9**│ **Fixation does not impact the quantity of HC bundles.** Two independent experiments of HC bundle staining (A-B and C-D, respectively) were repeated to examine the impact of fixation on the quantity of HC bundles observed in the 32-37 mm and 39-40 mm TL fish groups (n = 4 in each group). Scale bars: 5 μm (A-D).

**Supplementary** Table S1 Summary of AEP thresholds (Mean ± SEM, dBw).

| **F (Hz) 12-15 mm 17-20 mm 22-26 mm 32-37 mm 42-46 mm** |
| --- |
| 100 140.00 ± 4.73 131.25 ± 2.39 130.00 ± 3.53 125.00 ± 2.11 130.45 ± 1.25  200 134.00 ± 5.00 127.50 ± 2.50 125.00 ± 3.53 120.63 ± 2.40 126.36 ± 1.36  400 127.50 ± 4.77 121.25 ± 2.39 120.00 ± 3.53 116.25 ± 2.45 120.91 ± 1.48  600 120.00 ± 2.86 113.75 ± 3.15 111.25 ± 4.27 104.38 ± 2.58 110.00 ± 1.65  800 123.00 ± 2.88 115.00 ± 3.54 111.25 ± 3.15 106.88 ± 1.86 110.45 ± 1.57  1,000 124.00 ± 3.06 117.50 ± 2.50 113.75 ± 4.27 106.25 ± 2.06 112.73 ± 0.75  2,000 130.00 ± 2.39 128.75 ± 3.15 123.75 ± 4.27 117.50 ± 1.89 124.58 ± 1.29  3,000 129.00 ± 3.00 136.25 ± 3.75 127.50 ± 3.23 120.00 ± 2.50 131.25 ± 0.65  4,000 130.00 ± 3.33 127.50 ± 1.44 121.25 ± 3.29 120.00 ± 1.33 127.08 ± 1.30  6,000 132.00 ± 4.23 120.00 ± 0.00 120.00 ± 3.54 121.67 ± 1.67 128.75 ± 1.86  8,000 147.00 ± 2.58 133.75 ± 1.25 136.25 ± 3.75 135.00 ± 1.67 144.58 ± 1.56 |

F = frequency

**Supplementary Table S2 Information about the electrodes used**.

| **Electrodes** | **Catalogue number** | **Manufacturer** | **Detectable frequencies** |
| --- | --- | --- | --- |
| Tungsten electrodes | 563410 | A-M SYSTEMS/USA | ~12,000 Hz |
| Tungsten microelectrodes | 575300 | A-M SYSTEMS/USA | ~12,000 Hz |
| Tungsten microelectrodes | WE30010.01F5 (9-11 k) | Microprobe/USA | ~12,000 Hz |
| Tungsten microelectrodes | WE30010.1F5 (90-110 k) | Microprobe/USA | ~12,000 Hz |
| Silver electrodes | 786500 | A-M SYSTEMS/USA | ~8,000 Hz |
| Concentric bipolar electrodes | CBDMX75 (CW1) | FHC/USA | ~8,000 Hz |
